# Supplementary material for: A systematic review and evaluation of Zika virus forecasting and prediction research during a public health emergency of international concern
Source: PLoS Negl Trop Dis. 2019 Oct 4;13(10):e0007451. doi: 10.1371/journal.pntd.0007451 (PMC6805005; doi:10.1371/journal.pntd.0007451)
Supplement: S1 Table — (DOCX) [file pntd.0007451.s002.docx]

| **Table S1. Data abstraction and study evaluation tool used by reviewers** |
| --- |
| Lead author surname and first initial **(e.g Smith, J)** |
| How was the model published **(non-journal website only / journal / other (specify))** |
| If the model/forecast was published in a journal, was it made available as a pre-print (e.g Biorxiv) or as an rapid fast-tracked journal publication **(Y/N/can’t judge).** If Yes, what was date of pre-print?( **MM-DD-YYYY)** |
| If the model/forecast was published in a journal, please indicate which journal it was published in **(indicate NA if not published in a journal)** |
| Was the publication open access **(Y/N/can’t judge)** |
| Please indicate if any authors were affiliated with US Govt **(Y/N/can't judge)** |
| Please indicate if any authors were affiliated with Foreign Govt **(Y/N/can't judge)** |
| Please indicate if any authors were affiliated with academia **(Y/N/can't judge)** |
| Please indicate if any authors were affiliated with industry **(Y/N/can't judge)** |
| Please indicate if any authors were affiliated with any NGO **(Y/N/can't judge)** |
| Please indicate if any authors were affiliated with any other type of organization (e,g. WHO) **(please specify or indicate if can't judge)** |
| Study submission date **(MM-DD-YYYY or mark NA if not available)** |
| Study acceptance date **(MM-DD-YYYY or mark NA if not available)** |
| Study publication date **(MM-DD-YYYY or mark NA if not available)** |
| Date of most recent data point **(MM-DD-YYYY or MM-YYYY or mark NA if not applicable)** |
| Study funder(s) **(specify funder and award number, mark NA if unavailable)** |
| Was relevant data made available **(entirely / not at all / partially / can’t judge)** |
| Was relevant statistical modeling computational code made available, e.g. an R script **(entirely / not at all / partially / can’t judge)** |
| Were forecast/model methods presented with a level of detail that allowed the study to be reproduced **(entirely / not at all / partially / can’t judge)** |
| What was the model type **(please select which terms apply: stochastic, deterministic, spatial model, agent-based, mean-field, Bayesian hierarchial, machine learning, other please specify)** |
| Please describe the model used in your own words |
| Which data sources were used in the model **(e.g. case count data, Google data, genomic data, climate data, other please specify)** |
| Which Zika-related phenomenon were forecasted or predicted (**please select from the following: predicted microcephaly burdens, predicted Gullain-Barre burden, ZIKV disease peak size, peak timing, spatial spread, case fatality ratio, Ro, epidemic curve trajectory, epidemic final size, intervention impact, cost-effectiveness, force of infection, other please specify)** |
|  |
| In which geographic region were the models/forecasting systems making ZIKV predictions **(e.g. CONUS, Brazil, global, other please specify)?** |
| Is there a clear and accurate visual display of the model output **(entirely / not at all / partially / unable to judge)** |
| Were estimates of uncertainty (e.g confidence intervals) provided with model predictions? **(entirely/not at all/partially/can't judge)** |
